# The cost of cancer – A comparative analysis of the direct medical costs of cancer and other major chronic diseases in Europe

**DOI:** 10.1371/journal.pone.0241354

**Published:** 2020-11-11

**Authors:** Max Schlueter, Katie Chan, Romain Lasry, Martin Price

**Affiliations:** 1 Real World Solutions, IQVIA, London, United Kingdom; 2 Technology & Services, IQVIA, London, United Kingdom; 3 Janssen-Cilag, High Wycombe, Buckinghamshire, United Kingdom; Walter Sisulu University, SOUTH AFRICA

## Abstract

**Background:**

Cancer poses a significant mortality, morbidity, economic and humanistic burden to patients and health systems. This study aims to better understand healthcare expenditure on cancer relative to other major chronic diseases across France, Germany, Italy, Spain and the United Kingdom, whilst also considering the burden of illness posed by these conditions.

**Methods:**

A targeted literature review was performed to identify and extract relevant demographic, epidemiological and economic data. A health care payer perspective was adopted for the analysis, with a focus on direct healthcare costs.

**Results:**

Between 2006–2015, the cancer-related disability-adjusted life year (DALY) disease burden decreased by 9.3% despite a 6.5% increase in prevalence. Whilst the per patient drug costs increased by a compound annual growth rate (CAGR) of 5.1%, the overall per patient cancer costs decreased over the 10-year study period (CAGR of -1.4%). Compared to cardiovascular disease, neurological/mental disorders and diabetes, cancer was associated with the highest disease burden (20.8% of DALYs across all diseases) but the second-lowest healthcare expenditure levels (4.8% of total healthcare expenditure) among the studied major chronic diseases.

**Conclusions:**

Our study suggests that the costs associated with treating cancer account for a low proportion of total healthcare expenditure relative to the burden of the disease and compared to other major chronic diseases across the countries included in the analysis.

## Background

Major noncommunicable diseases including cancer, cardiovascular diseases, neurological disorders and diabetes are the leading cause of mortality and morbidity in Europe, accounting for 86% of all-cause mortality [1]. For cancer, 3.91 million new cases and 1.93 million deaths were estimated in Europe in 2018 [2]. The increase in cancer incidence and mortality is largely attributed to population growth, an ageing population and increased exposure to risk factors [3], resulting in a high unmet need for effective and well tolerated treatments as well as earlier disease detection.

Health care expenditure accounts for almost 10% of total spending in the European Union (EU) [4]. Constrained healthcare budgets have led to increasing pressure on payers to implement cost saving strategies [5].

While the estimated costs of cancer management are well described [3, 6–8], consideration of these costs in the context of the disease burden–as captured by the DALY metric–is relevant and important. Furthermore, comparisons of the economic and disease burden between the major chronic diseases may facilitate health policy and decisions regarding resource allocation.

The objective of this study was to compare the burden of cancer relative to other major chronic diseases and associated health care expenditures in France, Germany, Italy, Spain and the United Kingdom. These countries were selected for the analysis as they constitute the largest countries by population in the European Union.

## Methods

Four chronic diseases accounting for the majority of disease burden (as measured by DALY) in Europe were considered: cancer, cardiovascular disease (CVD), neurological/mental disorders and diabetes [9]. Cancer was defined as per the World Health Organisation (WHO) International Classification of Diseases, 10^th^ revision, codes C00-D48 (all neoplasms) [10]. Cardiovascular disease also included chronic heart disease (CHD) and stroke (ICD-10 codes I00-I99). Diabetes comprised both type 1 and type 2 diabetes mellitus (E10-E11). Neurological disorders included Alzheimer disease and other dementias (ICD-10 code F00-F03), epilepsy (G40), multiple sclerosis (G35) and Parkinson disease (G20).

A targeted review of the published and grey literature was conducted to identify relevant demographic, epidemiological and economic data. Searches were performed in PubMed and Google Scholar. For the demographic search, the search terms “population age structure” AND “Europe” were applied. The terms “prevalence” AND “DALY” AND “disease term” were used for the epidemiology search, whereas for the economic data search, the search terms “cost” AND/OR “burden” AND “disease term” AND “Europe” were applied. A range of data sources were considered for the analysis, including international bodies and national databases as well as reports and publications from individual countries [11–26]. A systematic and hierarchical selection of sources was adopted whereby preference was given to international sources from well-established organizations such as the World Health Organization (WHO), the Organization for Economic Cooperation and Development (OECD), and the Statistical Office of the European Communities (EUROSTAT) for the final analyses given that the screening of individual country sources revealed inconsistent methods to collect and report data. Owing to data availability limitations beyond 2015 for some of the required data, the 10-year study period covered years 2006 to 2015.

### Demographic data

Country-specific data comprised estimates of the population age structure (both sexes combined, thousands) for each of the included countries. These were derived from the 2017 report of the United Nations, “World Population Prospects” [27].

### Epidemiology data

For each country, prevalence, incidence, cause of death, and disability-adjusted life year (DALY) estimates were extracted for each disease from the Global Burden of Diseases results tool [28]. The Global Burden of Diseases, Injuries, and Risk Factors Study (GBD) is the single largest and most detailed scientific effort conducted to quantify levels and trends in global health, led by the Institute for Health Metrics and Evaluation (IHME). The GBD Study 2016 complied with the Guidelines for Accurate and Transparent Health Estimates Reporting (GATHER) and had participation from over 1,000 researchers from more than 100 countries, including 26 low- and middle-income countries.

Prevalence and incidence rates in GBD are informed by multiple sources for each country and estimated using DisMod-MR 2.1, a Bayesian meta-regression tool [29]. Cause-specific deaths by age, country, and year were derived from the GBD cause of death database [30]. DALY estimates were extracted from the GBD results tool, Global Health Data Exchange (GHDx), which uses results from the Global Burden of Diseases, Injuries, and Risk Factors Study 2017 for all-cause mortality, cause-specific mortality, and non-fatal disease burden to derive DALYs; these were calculated by summing years of life lost and years of life lived with disability for each location, age group, sex, and year [9]. All GBD data were extracted as absolute values for all ages and as age-standardised rate per 100,000.

Default settings applied for extraction of data from GHDx are provided in S1 Table.

### Economic data

Economic data included total healthcare expenditure, total disease direct costs, and disease drug cost estimates for each of the included countries. Total healthcare expenditure was defined as the overall spending on healthcare goods and services for all diseases at a country level, whereas total disease direct costs and disease drug cost refer respectively to total direct costs and drug costs attributed to a specific disease. Only direct costs were considered given the study objective of estimating the economic burden to healthcare payers that impact decision-making on resource allocation. All economic data are expressed in million Euros (converted at purchasing power parity when necessary) and were inflated to 2015 values [31]. Any estimates in currencies other than Euros were converted using the OECD exchange rate index [32].

Total healthcare expenditure for each country was sourced from the 2017 edition of the System of Health Accounts (SHA) developed by the WHO and the OECD to calculate health expenditures in a harmonised way [33]. The SHA framework considers the final consumption of health goods and services from public and private sources. To estimate total healthcare expenditure per country of interest, the country’s per capita healthcare expenditure was multiplied by the country population estimates described above.

To ensure the most cross-country comparisons possible, international sources that offered the most complete data and consistent methodologies across countries were used for each disease area. Estimates of the direct cost of cancer, which comprises expenditures for primary prevention measures, screening programs, diagnosis, pharmacological and non-pharmacological treatment, rehabilitation and palliative care, were sourced from reports of the Swedish Institute for Health Economics (IHE) [3, 6]. Direct cost of CVD was informed by estimates from the European Heart Network [34], whereas estimates of direct cost of diabetes were derived from a range of editions from the Diabetes Atlas published by the International Diabetes Foundation (IDF) [35]. For neurological disorders, direct costs for specific disease areas were estimated from publications [36, 37].

Annual drug expenditure was extracted from the IQVIA MIDAS database, an analytics platform capturing over 94% of the global prescription and pharmaceutical sales universe [38]. The Anatomical Therapeutic Chemical (ATC) classification was used to determine inclusion in the list of pharmaceutical products for each therapeutic area. The list of ATC codes per disease area is provided in S1 Fig. Prescription and sales data from retail and hospital settings were summed to derive the total drug expenditure estimates for each disease area.

The methodological framework for analysis generally follows that of established cost of illness studies [39, 40]. For expenditure estimates mentioned above, either the overall percentage change or the compound annual growth rate (CAGR) was calculated to elicit trends over the ten-year study period, the latter of which provides a representation of the mean annual growth rate over a specified period of time. CAGR was selected as it provides a measure of growth over multiple time periods that takes into account compounding over the overall time period [41]. Per patient expenditure on each disease was calculated by dividing disease specific expenditure by the disease specific prevalence for each country. The proportion of disease specific expenditure or DALY disease burden was calculated by dividing the disease specific expenditure or DALY disease burden by the total healthcare expenditure or total DALY disease burden. In cases of missing data from the consulted sources, estimates were interpolated linearly.

Country-level data inputs used for the analysis are presented in S2 Table.

## Results

**Fig 1:**
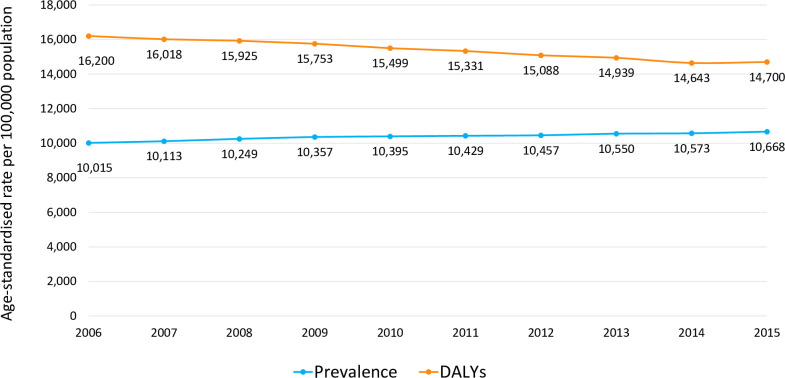
Cancer prevalence and DALYs lost due to cancer in France, Germany, Italy, Spain, UK (2006–2015). Age-standardised rate (ASR): a summary measure of the rate that a population would have if it had a standard age structure. Prevalence: number of current cases (new and pre-existing) at a specified time. Disability-adjusted life year (DALY): measure combining the burden of mortality and disability, calculated as the sum of the Years of Life Lost (YLL) due to premature mortality in the population and the Years Lost due to Disability (YLD) for people living with the health condition or its consequences.

The prevalence of cancer in the included countries increased by 6.5% between 2006 and 2015, with the all age-standardised rate (ASR, per 100,000 persons) rising from 10,015 to 10,668. Over the same period, the cancer-related DALY disease burden decreased by 9.3% from 16,200 to 14,700 DALYs (Fig 1).

**Fig 2:**
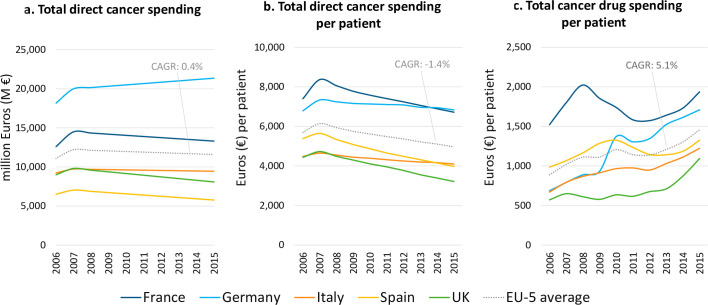
Cancer expenditure by country (2006–2015).

Total direct costs of cancer in the included countries were estimated to amount to €58.0 billion in 2015, accounting for 4.8% of total health care expenditures. Fig 2A shows that expenditure was highest in Germany (€21.4 billion), followed by France (€13.3 billion), Italy (€9.4 billion), UK (€8.1 billion) and Spain (€5.8 billion). Total cancer expenditure levels changed marginally over the period 2006–15 (CAGR of 0.4%). As Fig 2B illustrates, direct per patient expenditures decreased over the 10-year study period (CAGR of -1.4%). In 2015, they averaged €4,966, being highest in Germany (€6,833) and France (€6,718), and lowest in UK (€3,211). Total cancer drug spending per patient suggested an increasing trend over the study period, growing at a CAGR of 5.1% and averaging €1,457 in 2015 across the included countries (Fig 2C).

**Fig 3:**
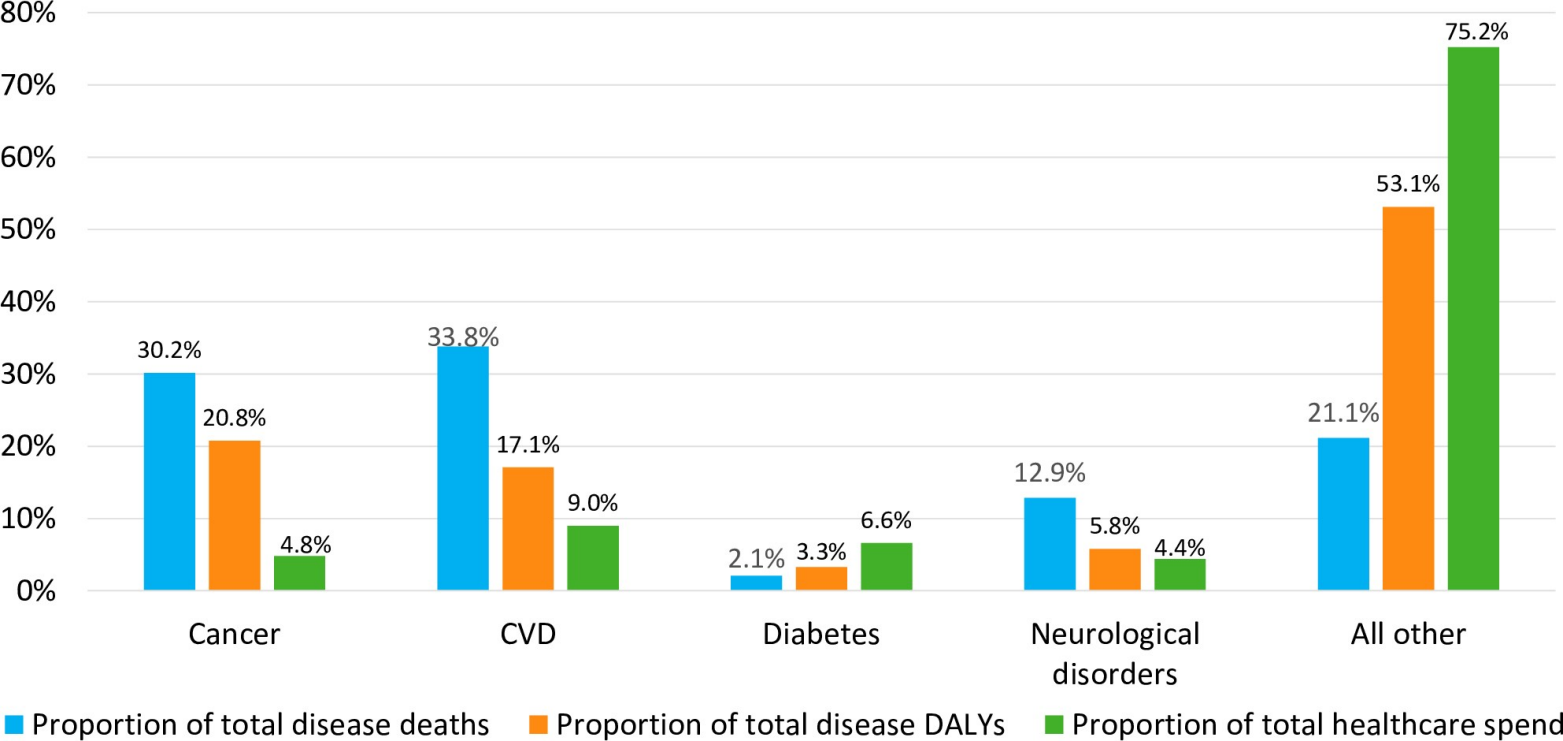
Average proportion of mortality, DALYs and healthcare expenditure, by disease (2015).

To better understand the context of spending, we compared cancer to the other leading causes of mortality in Europe. Among the major chronic diseases, cancer imposed the highest DALY disease burden across France, Germany, Italy, Spain and the United Kingdom, amounting to 20.8% of total DALYs for all diseases combined, followed by CVD, neurological disorders and diabetes. Cancer (30.2%) and CVD (33.8%) together accounted for about two thirds of all disease-related deaths. The highest proportion of healthcare spending was attributed to CVD (9.0%), followed by diabetes, cancer and neurological disorders. Whilst healthcare expenditure in the included countries was lower than the relative proportions of disease-related deaths and total DALYs among cancer, CVD and neurological disorders, the inverse was true for diabetes, where healthcare expenditure (6.6%) was triple the proportion of deaths (2.1%) (Fig 3).

**Fig 4:**
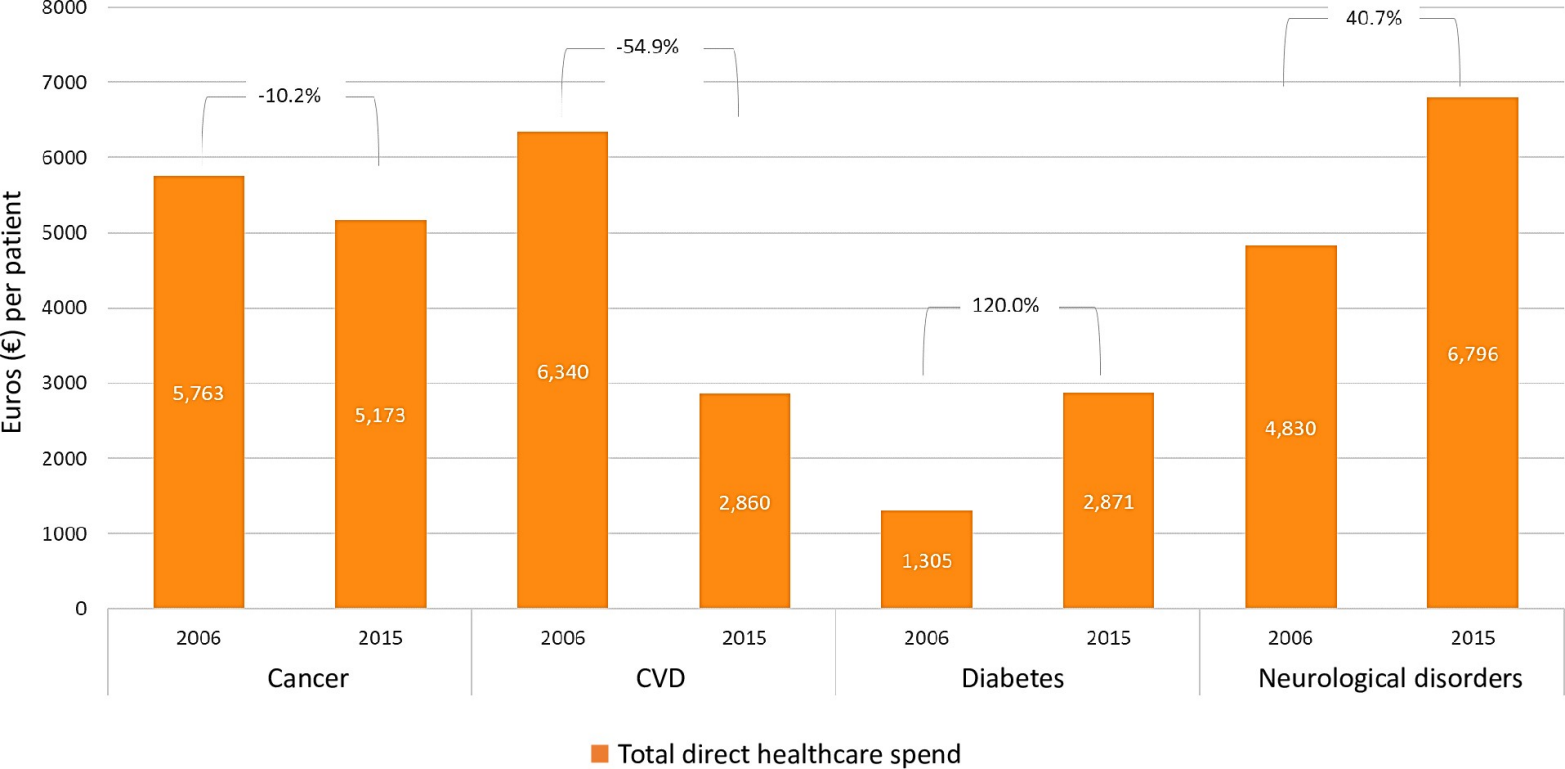
Total direct healthcare expenditure per patient, by disease (2006–2015). Percentage estimates denote percent change in total direct healthcare expenditure per patient between 2006 and 2015 in each disease area.

While the total annual direct cancer expenditure per patient decreased over the 10-year study period (-10.2%), substantial increases were observed for diabetes (120.0%) and neurological disorders (40.7%), and a substantial decrease for cardiovascular disease (-54.9%) (Fig 4). In absolute terms, direct medical costs per patient were highest for neurological disorders, followed by cancer.

In terms of overall healthcare costs across the countries included in the analysis, cancer was associated with the second-lowest proportion of total direct healthcare expenditure (4.8%), compared to 4.4% for neurological disorders, 6.6% for diabetes and 9.0% for CVD (Fig 3). Cancer is therefore associated with the lowest healthcare expenditure to DALY disease burden and mortality ratio among the major chronic diseases.

## Discussion

This study set out to achieve a better understanding of the relationship between direct health spend on cancer relative to other major chronic diseases (versus total healthcare spend) together with a comparison of the associated burden of disease in France, Germany, Italy, Spain and the United Kingdom. Data underpinning our analysis was elicited from a wide range of sources that were identified and selected with particular consideration of data quality and comparability across countries and therapeutic areas.

The DALY burden of cancer fell by 9.3% between 2006 and 2015 despite an increase of 6.5% in cancer prevalence over the same period. Further research is warranted to establish causal factors for this association, which are likely to include advances in screening and disease detection since earlier detection increases the likelihood of successful treatment.

Drug costs were found to account for about a third of the direct total costs of cancer care per patient (2015 figures). Despite an increasing trend amounting to a CAGR of 5.1% (period 2006–15) in per patient cancer drug expenditure, overall direct cancer costs per patient decreased (CAGR of -1.4%). Again, further research is needed to identify the factors underlying this trend, which may include improved patient outcomes due to earlier disease detection, advances in precision and personalised medicine, and corresponding offsets in healthcare resource utilisation.

Compared to the other major chronic diseases examined, cancer was associated with the highest DALY burden (20.8% of the total) and the second highest mortality burden (30.2% of the total) in 2015. By contrast, cancer accounted for only 4.8% of total healthcare expenditure, the second-lowest level of all studied major chronic diseases. Additionally, although cancer continues to incur the second-highest per patient cost in absolute terms, comparison of total direct per patient expenditures across the major chronic diseases showed these to decrease in cancer whereas significant increases were observed for diabetes and neurological disorders.

These findings may be informative in a policy context of determining optimal healthcare funding across the major chronic diseases. Considerations of healthcare resource allocation follow different distributive principles, commonly categorised as utilitarian, egalitarian and prioritarian [42, 43]. From a prioritarian or needs-based perspective, the high mortality and DALY burden imposed by cancer would warrant a shift in expenditure levels towards this therapy area (e.g. for screening programs, molecular testing, disease management, affordable precision medicines). Implications from an egalitarian perspective would depend on the maxim applied: if seeking to equalise health outcomes the distributive implications would be similar to the prioritarian principle, whereas a focus on fair equality of opportunity for health would entail an emphasis on egalitarian access to health care and health promotion programs [44, 45]. Rationing decisions from a utilitarian perspective in healthcare commonly adopt cost-effectiveness analysis (CEA) to ascertain which health technologies should be endorsed to maximise aggregate population health. Even in the UK, however, which has long adopted CEA, introduction of policies such as the Highly Specialised Technology programme epitomises the reluctance to invariably adopt the utilitarian maxim of aggregating health benefits across the population, particularly for diseases with a very high disease burden [46, 47]. Besides these distributive principles, mechanisms of financing healthcare in the included EU countries [48] are also likely to impact each country’s approach to resource allocation across the studied therapy areas.

A number of limitations of the analysis are noted that may have influenced the findings. First, the validity and precision of our findings is dependent on the availability and quality of robust, comparable data across EU countries. However, efforts to systematically and routinely collect data–particularly for overall expenditure–in a standardised way for major disease areas are not consistent across European countries. Our results may thus have been impacted by differences in data format, completeness and quality across included countries, which partly may be due to differing healthcare systems. For example, in decentralised healthcare systems such as Italy and Spain, aggregation and collection of national level data is limited or not routinely performed. Use of established single data sources for epidemiological data (GHDx) and drug costs (MIDAS) for this study was intended to improve comparability, however, the outputs from these databases themselves make use of assumptions and extrapolations.

Data on expenditure trends over time may have been impacted by wider economic policy, developments or shocks, that affect the ability of European governments to fund health care, such as the global financial crisis in 2009. The main impact of macroeconomic constraints is likely to be on overall healthcare expenditure, and comparisons of different cost constituents and across therapeutic areas are less likely be affected. Expenditure trends in the therapeutic areas analysed for this study may, however, have been driven by specific health care reforms, the impact of which will need to be assessed in future research. Similarly, further research is necessary to identify causal factors–and quantify their impact–effecting DALY decreases in oncology despite increases in prevalence, for example the upscaling of early screening programs, molecular testing or improved therapeutic options.

The estimates presented in this study are underestimates of the overall societal economic burden as only direct costs were considered. Adoption of a societal perspective that takes into account informal care costs and productivity losses due to absenteeism from work, early retirement or impact on caregivers would substantially add to the economic burden, as suggested by a population-based cost analysis of the economic burden of cancer in the EU [7].

Conversely, the direct drug cost estimates presented in our study may overstate the true economic burden to health care payers given that mechanisms such as confidential value-based pricing and managed entry agreements typically result in acquisition costs below that of public list prices [49, 50].

The study did not longitudinally analyse costs accruing over an average patient lifetime, therefore potentially underestimating the overall cost associated with chronic diseases with a low mortality burden, such as diabetes. The analysis does, however, consider direct per patient cost based on prevalent patient populations, which captures the mortality burden within the analysis period.

Finally, some costs may have been double-counted as complications relating to one therapy area (e.g. diabetes) may fall into another of the investigated therapy areas (e.g. peripheral vascular disease, stroke or myocardial infarction as cardiovascular conditions/events). The extent of such potential double-counting is challenging to gauge and an area for future research.

Despite the study limitations, we believe that our study is of value to decision makers in Europe, as to our knowledge this is the first study to comparatively assess the economic and DALY disease burden of cancer with that of other major chronic diseases in these countries. Our study suggests that healthcare costs associated with cancer care account for a low proportion of total healthcare expenditure relative to the burden of the disease in France, Germany, Italy, Spain and the United Kingdom, and relative to expenditure levels in other major chronic diseases.

## Supporting information

S1 Fig Market definition of drugs by ATC codes. (DOCX)

S1 Table Default settings used for retrieval of data from GHDx. (DOCX)

S2 Table Data inputs table. (DOCX)
