# Supplementary figures and images for: The cost of cancer – A comparative analysis of the direct medical costs of cancer and other major chronic diseases in Europe
Source: PLoS One. 2020 Nov 11;15(11):e0241354. doi: 10.1371/journal.pone.0241354 (PMC7657541; doi:10.1371/journal.pone.0241354)

S1 Fig. Market definition of drugs by ATC codes


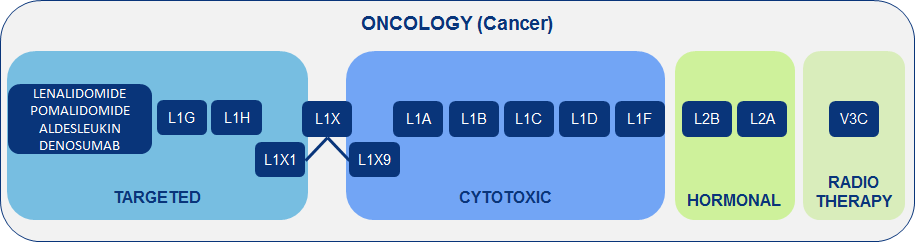


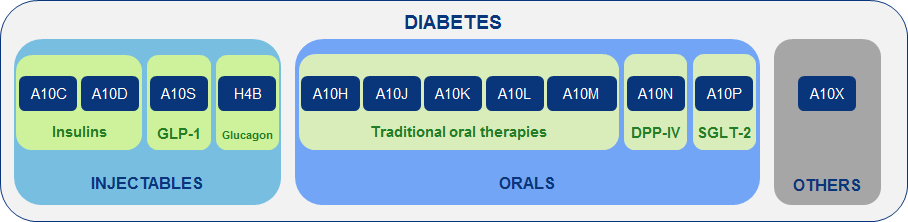


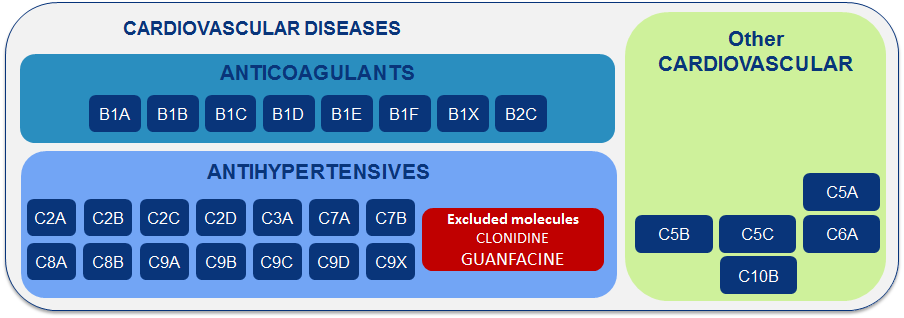


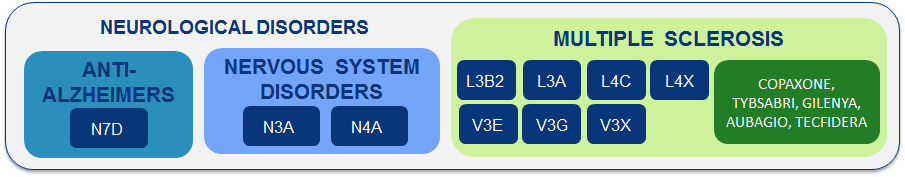

Supplement: S1 Fig — (DOCX) [file pone.0241354.s001.docx]
