# Supplementary material for: The cost of cancer – A comparative analysis of the direct medical costs of cancer and other major chronic diseases in Europe
Source: PLoS One. 2020 Nov 11;15(11):e0241354. doi: 10.1371/journal.pone.0241354 (PMC7657541; doi:10.1371/journal.pone.0241354)
Supplement: S1 Table — (DOCX) [file pone.0241354.s002.docx]

S1 Table. Default settings used for retrieval of data from GHDx

| **Variable** | **Default settings** | |
| --- | --- | --- |
| Sex | Both | |
| Age | For rates: age-standardised  For absolute numbers: all ages, otherwise specified age range | |
| Cause | All diseases | A. Communicable, maternal, neonatal, and nutritional diseases  B. Non-communicable diseases |
|  | Cancers | B.1 Neoplasms |
|  | Cardiovascular diseases | B.2 Cardiovascular diseases |
|  | Diabetes | B.8.1 Diabetes mellitus |
|  | Neurological disorders | B.6.1 Alzheimer disease and other dementias  B.6.2 Parkinson disease  B.6.3 Epilepsy  B.6.4 Multiple sclerosis |

Assumptions: Prevalence (absolute number), was assumed to be the number of persons with that cause in the specified time period. i.e. cancer prevalence (absolute number, all ages) in 2017, was assumed to be the cancer population in 2017.
