# Supplementary material for: The cost of cancer – A comparative analysis of the direct medical costs of cancer and other major chronic diseases in Europe
Source: PLoS One. 2020 Nov 11;15(11):e0241354. doi: 10.1371/journal.pone.0241354 (PMC7657541; doi:10.1371/journal.pone.0241354)
Supplement: S2 Table — (DOCX) [file pone.0241354.s003.docx]

**S2 Table. Data inputs table**

| **Prevalence (absolute)** | | | | | | | | | | |  |
| --- | --- | --- | --- | --- | --- | --- | --- | --- | --- | --- | --- |
| **Cancer** | **2006** | **2007** | **2008** | **2009** | **2010** | **2011** | **2012** | **2013** | **2014** | **2015** | **Source** |
| France | 1700701 | 1731672 | 1779212 | 1825110 | 1853218 | 1876501 | 1898803 | 1926264 | 1952284 | 1980504 | Global Burden of Disease Study 2017 (GBD 2017) |
| Germany | 2672020 | 2725520 | 2783559 | 2838809 | 2874337 | 2908972 | 2942767 | 3017608 | 3050549 | 3125635 |  |
| Italy | 2058536 | 2094779 | 2131965 | 2175531 | 2193654 | 2223567 | 2249329 | 2268058 | 2277363 | 2308453 |  |
| Spain | 1207047 | 1246683 | 1288469 | 1321894 | 1347467 | 1374984 | 1392225 | 1409421 | 1427476 | 1449454 |  |
| UK | 2026597 | 2074604 | 2137975 | 2181980 | 2230725 | 2263419 | 2316747 | 2396050 | 2450546 | 2517316 |  |
| **CVD** |  |  |  |  |  |  |  |  |  |  |  |
| France | 6579029 | 6680509 | 6786833 | 6898792 | 7013288 | 7134964 | 7261752 | 7394333 | 7530766 | 7674373 | Global Burden of Disease Study 2017 (GBD 2017) |
| Germany | 10299954 | 10382761 | 10461531 | 10553192 | 10642362 | 10728788 | 10811691 | 10897561 | 10997621 | 11080633 |  |
| Italy | 6714169 | 6842152 | 6971724 | 7095161 | 7193944 | 7270783 | 7339065 | 7392454 | 7439597 | 7467812 |  |
| Spain | 4253657 | 4308092 | 4361443 | 4420235 | 4478087 | 4547967 | 4602966 | 4658897 | 4715194 | 4764009 |  |
| UK | 6347873 | 6412105 | 6485495 | 6561293 | 6640374 | 6713743 | 6798958 | 6883931 | 6963648 | 7039448 |  |
| **Diabetes** |  |  |  |  |  |  |  |  |  |  |  |
| France | 3568757 | 3693645 | 3825023 | 3951948 | 4064623 | 4166877 | 4268609 | 4369693 | 4470069 | 4567154 | Global Burden of Disease Study 2017 (GBD 2017) |
| Germany | 6130899 | 6303556 | 6500619 | 6698196 | 6855995 | 6980552 | 7116236 | 7262319 | 7422858 | 7586690 |  |
| Italy | 4824067 | 4906076 | 4992958 | 5087656 | 5191378 | 5307965 | 5439240 | 5586350 | 5752169 | 5931936 |  |
| Spain | 3483782 | 3566984 | 3656715 | 3747310 | 3826787 | 3902271 | 3969180 | 4034927 | 4099693 | 4159555 |  |
| UK | 4120094 | 4242553 | 4379206 | 4521550 | 4663368 | 4811266 | 4981790 | 5170032 | 5374060 | 5591745 |  |
| **Neurological disorders** | | | | | | | | | | |  |
| France | 1243582 | 1269117 | 1293982 | 1317587 | 1342934 | 1372126 | 1402799 | 1435014 | 1468493 | 1502554 | Global Burden of Disease Study 2017 (GBD 2017) |
| Germany | 1932800 | 1959198 | 1983536 | 2017673 | 2056917 | 2090574 | 2118597 | 2145631 | 2180268 | 2212540 |  |
| Italy | 1286238 | 1322355 | 1356124 | 1388014 | 1418824 | 1452220 | 1479209 | 1504094 | 1529641 | 1551992 |  |
| Spain | 928564 | 945768 | 960545 | 975174 | 989415 | 1008113 | 1020504 | 1029786 | 1037091 | 1037488 |  |
| UK | 1156362 | 1171931 | 1186798 | 1202047 | 1218471 | 1233808 | 1248022 | 1260590 | 1272904 | 1285302 |  |
|  |  |  |  |  |  |  |  |  |  |  |  |
| **DALYs (absolute)** | | | | | | | | | | |  |
| **Cancer** | **2006** | **2007** | **2008** | **2009** | **2010** | **2011** | **2012** | **2013** | **2014** | **2015** | **Source** |
| France | 3364400 | 3355521 | 3388219 | 3438911 | 3431887 | 3438045 | 3426854 | 3412225 | 3383293 | 3397749 | Global Burden of Disease Study 2017 (GBD 2017) |
| Germany | 4467560 | 4488363 | 4537744 | 4586710 | 4591055 | 4593282 | 4568088 | 4651236 | 4580501 | 4703606 |  |
| Italy | 3283435 | 3297235 | 3322016 | 3333605 | 3287791 | 3319107 | 3323773 | 3289508 | 3253308 | 3315795 |  |
| Spain | 2099720 | 2141062 | 2165128 | 2163071 | 2167055 | 2193904 | 2193434 | 2179460 | 2180627 | 2214819 |  |
| UK | 3169159 | 3156953 | 3190205 | 3162985 | 3170310 | 3135218 | 3133886 | 3188709 | 3190530 | 3256061 |  |
| **CVD** |  |  |  |  |  |  |  |  |  |  |  |
| France | 2225998 | 2195792 | 2198428 | 2206384 | 2180678 | 2166296 | 2155613 | 2134183 | 2102077 | 2127796 | Global Burden of Disease Study 2017 (GBD 2017) |
| Germany | 4876440 | 4812660 | 4783192 | 4781278 | 4723184 | 4663533 | 4612097 | 4678644 | 4577687 | 4731244 |  |
| Italy | 2770844 | 2758006 | 2756500 | 2753682 | 2689756 | 2715454 | 2717377 | 2673088 | 2641220 | 2719623 |  |
| Spain | 1826055 | 1822254 | 1797947 | 1744924 | 1704425 | 1703886 | 1694994 | 1662981 | 1661824 | 1706109 |  |
| UK | 3028281 | 2920847 | 2866205 | 2749310 | 2678821 | 2585659 | 2545669 | 2564496 | 2540723 | 2589192 |  |
| **Diabetes** |  |  |  |  |  |  |  |  |  |  |  |
| France | 373740 | 378395 | 385940 | 393238 | 396422 | 400052 | 404186 | 407149 | 407149 | 416544 | Global Burden of Disease Study 2017 (GBD 2017) |
| Germany | 615788 | 626588 | 642961 | 661785 | 673382 | 678193 | 682804 | 695869 | 695869 | 717796 |  |
| Italy | 617717 | 626181 | 634253 | 643813 | 646623 | 659670 | 671230 | 679150 | 679150 | 712923 |  |
| Spain | 350583 | 356348 | 360915 | 362573 | 363738 | 367008 | 368643 | 369076 | 369076 | 378990 |  |
| UK | 350862 | 355434 | 363177 | 368746 | 376167 | 382772 | 392459 | 405482 | 405482 | 434394 |  |
| **Neurological disorders** | | | | | | | | | | |  |
| France | 776548 | 790317 | 810335 | 829841 | 843056 | 862349 | 879649 | 890956 | 899423 | 913467 | Global Burden of Disease Study 2017 (GBD 2017) |
| Germany | 1095534 | 1118883 | 1144612 | 1175042 | 1203210 | 1224353 | 1232583 | 1255847 | 1265011 | 1304256 |  |
| Italy | 776489 | 799054 | 820587 | 841115 | 852933 | 878835 | 899206 | 909317 | 923417 | 955585 |  |
| Spain | 571027 | 585061 | 596631 | 602567 | 608454 | 622669 | 630219 | 634771 | 643444 | 655532 |  |
| UK | 750333 | 760018 | 775093 | 780229 | 792622 | 802129 | 819284 | 839134 | 850075 | 874871 |  |
| **All disease areas** |  |  |  |  |  |  |  |  |  |  |  |
| France | 14334844 | 14355582 | 14490800 | 14640407 | 14662848 | 14697996 | 14734442 | 14743923 | 14696857 | 14816343 | Global Burden of Disease Study 2017 (GBD 2017) |
| Germany | 22544147 | 22568171 | 22647673 | 22778883 | 22786782 | 22758671 | 22693368 | 22973778 | 22825208 | 23322547 |  |
| Italy | 14977131 | 15063252 | 15174605 | 15266558 | 15186281 | 15325069 | 15393638 | 15333294 | 15287280 | 15530790 |  |
| Spain | 10540968 | 10678527 | 10747852 | 10722307 | 10711091 | 10785559 | 10785442 | 10719796 | 10742592 | 10867918 |  |
| UK | 16370153 | 16324903 | 16406776 | 16299609 | 16299450 | 16218014 | 16245781 | 16436528 | 16489226 | 16763185 |  |
| **Mortality (absolute)** | | | | | | | | | | |  |
| **Cancer** | **2006** | **2007** | **2008** | **2009** | **2010** | **2011** | **2012** | **2013** | **2014** | **2015** | **Source** |
| France | 165648 | 166658 | 169814 | 173315 | 174052 | 175839 | 177086 | 177545 | 177237 | 179484 | Global Burden of Disease Study 2017 (GBD 2017) |
| Germany | 217321 | 219894 | 224123 | 228514 | 230950 | 233541 | 234375 | 240187 | 238803 | 246737 |  |
| Italy | 170688 | 172955 | 175954 | 177859 | 176856 | 179986 | 181570 | 180654 | 180138 | 185073 |  |
| Spain | 103971 | 106586 | 108492 | 109173 | 110078 | 112461 | 113484 | 113378 | 114242 | 116762 |  |
| UK | 163311 | 163412 | 165792 | 164969 | 166057 | 165446 | 166948 | 170430 | 171107 | 175166 |  |
| **CVD** |  |  |  |  |  |  |  |  |  |  |  |
| France | 147133 | 146160 | 148031 | 149337 | 148038 | 148322 | 149304 | 148346 | 146672 | 150674 | Global Burden of Disease Study 2017 (GBD 2017) |
| Germany | 322233 | 319615 | 320656 | 324750 | 325730 | 326379 | 326762 | 335526 | 332057 | 348755 |  |
| Italy | 203243 | 204673 | 206577 | 208002 | 204671 | 209557 | 211599 | 208591 | 207489 | 217345 |  |
| Spain | 121879 | 122323 | 121720 | 118615 | 116330 | 117804 | 118584 | 117023 | 118207 | 123442 |  |
| UK | 197252 | 190833 | 187655 | 179555 | 175607 | 170442 | 169728 | 170806 | 168269 | 171796 |  |
| **Diabetes** |  |  |  |  |  |  |  |  |  |  |  |
| France | 10817 | 10726 | 10836 | 10892 | 10685 | 10587 | 10563 | 10397 | 10177 | 10387 | Global Burden of Disease Study 2017 (GBD 2017) |
| Germany | 17229 | 17102 | 17280 | 17683 | 17885 | 17877 | 17778 | 18156 | 17796 | 18672 |  |
| Italy | 16957 | 17334 | 17695 | 18073 | 17876 | 18379 | 18653 | 18455 | 18329 | 19208 |  |
| Spain | 8879 | 8974 | 8967 | 8713 | 8460 | 8423 | 8293 | 8027 | 8036 | 8315 |  |
| UK | 5633 | 5473 | 5437 | 5234 | 5172 | 5055 | 5058 | 5136 | 5102 | 5281 |  |
| **Neurological disorders** | | | | | | | | | | |  |
| France | 60173 | 61724 | 63997 | 65989 | 67474 | 69882 | 72103 | 73546 | 74745 | 76784 | Global Burden of Disease Study 2017 (GBD 2017) |
| Germany | 73506 | 76336 | 79421 | 83167 | 86906 | 89343 | 90057 | 92181 | 93962 | 98548 |  |
| Italy | 62495 | 64657 | 66601 | 68304 | 69458 | 72426 | 74619 | 75619 | 77235 | 81055 |  |
| Spain | 44881 | 46401 | 47815 | 48661 | 49541 | 51269 | 52492 | 53367 | 54781 | 56734 |  |
| UK | 55829 | 57021 | 58727 | 59636 | 61431 | 63139 | 65636 | 67839 | 69040 | 71822 |  |
| **All disease areas** |  |  |  |  |  |  |  |  |  |  |  |
| France | 480948 | 482833 | 492304 | 500938 | 502074 | 507288 | 512991 | 514002 | 512175 | 523422 | Global Burden of Disease Study 2017 (GBD 2017) |
| Germany | 773359 | 779689 | 792856 | 810981 | 822550 | 830935 | 834405 | 857028 | 853831 | 892542 |  |
| Italy | 539505 | 547513 | 556740 | 564239 | 561325 | 575783 | 583738 | 580548 | 581010 | 605026 |  |
| Spain | 363324 | 369828 | 373459 | 371304 | 370551 | 378288 | 382679 | 381499 | 386926 | 401201 |  |
| UK | 558249 | 553638 | 556631 | 547193 | 547043 | 543361 | 548733 | 558692 | 558055 | 573379 |  |
| **Total direct expenditure (million Euros)** | | | | | | | | | | | |
| **Cancer** | **2006** | **2007** | **2008** | **2009** | **2010** | **2011** | **2012** | **2013** | **2014** | **2015** | **Source** |
| France | 12586 | 14485 | 14338 | 14190 | 14043 | 13895 | 13748 | 13601 | 13453 | 13306 | IHE reports 2004, 2007, 2014, inflation-adjusted and with linear interpolation |
| Germany | 18141 | 19987 | 20158 | 20329 | 20501 | 20672 | 20844 | 21015 | 21186 | 21358 |  |
| Italy | 9226 | 9734 | 9698 | 9662 | 9626 | 9590 | 9554 | 9518 | 9482 | 9447 |  |
| Spain | 6497 | 7037 | 6878 | 6719 | 6560 | 6401 | 6242 | 6083 | 5924 | 5765 |  |
| UK | 8992 | 9805 | 9590 | 9374 | 9159 | 8944 | 8729 | 8513 | 8298 | 8083 |  |
| **CVD** |  |  |  |  |  |  |  |  |  |  |  |
| France | 31141 | 26509 | 21878 | 17247 | 17575 | 17903 | 18232 | 18560 | 18888 | 19216 | European CVD Statistics reports 2006, 2009, 2015, inflation-adjusted and with linear interpolation |
| Germany | 83768 | 71149 | 58531 | 45912 | 44716 | 43519 | 42323 | 41126 | 39930 | 38733 |  |
| Italy | 35281 | 30754 | 26226 | 21698 | 21687 | 21675 | 21664 | 21653 | 21642 | 21630 |  |
| Spain | 14056 | 13155 | 12253 | 11351 | 11460 | 11569 | 11678 | 11787 | 11896 | 12005 |  |
| UK | 52560 | 40280 | 27999 | 15719 | 15960 | 16201 | 16443 | 16684 | 16926 | 17167 |  |
| **Diabetes** |  |  |  |  |  |  |  |  |  |  |  |
| France | 5736 | 7763 | 9790 | 11817 | 13844 | 13635 | 13735 | 13836 | 15531 | 17226 | IDF Atlas reports 2007, 2010, 2011, 2013, 2015, inflation-adjusted and with linear interpolation |
| Germany | 14281 | 16435 | 18589 | 20743 | 22897 | 19387 | 23316 | 27244 | 29285 | 31325 |  |
| Italy | 4790 | 5839 | 6888 | 7937 | 8986 | 9516 | 9553 | 9590 | 10251 | 10912 |  |
| Spain | 1708 | 2624 | 3540 | 4456 | 5371 | 6995 | 8162 | 9330 | 9257 | 9184 |  |
| UK | 2796 | 3712 | 4627 | 5543 | 6458 | 10069 | 9577 | 9085 | 10178 | 11271 |  |
| **Neurological disorders** | | | | | | | | | | |  |
| France | 5226 | 6184 | 7142 | 8100 | 9058 | 10016 | 10974 | 11932 | 12890 | 13847 | Costs of Disorders of the Brain in Europe reports 2004, 2010, inflation-adjusted and with linear interpolation |
| Germany | 8082 | 8024 | 7965 | 7906 | 7848 | 7789 | 7731 | 7672 | 7614 | 7555 |  |
| Italy | 5691 | 5599 | 5506 | 5414 | 5321 | 5229 | 5136 | 5044 | 4951 | 4859 |  |
| Spain | 4694 | 5470 | 6247 | 7023 | 7799 | 8576 | 9352 | 10128 | 10905 | 11681 |  |
| UK | 7493 | 8364 | 9235 | 10106 | 10977 | 11848 | 12719 | 13590 | 14461 | 15332 |  |
| **All diseases** |  |  |  |  |  |  |  |  |  |  |  |
| France | 187781 | 177814 | 169870 | 191192 | 204249 | 199266 | 215399 | 219782 | 225960 | 270633 | Calculated from: a) OECD, Health spending, 2017. b) United Nations, World Population Prospects, 2017. Inflation-adjusted. |
| Germany | 262598 | 246423 | 237917 | 267082 | 289690 | 280349 | 305104 | 306471 | 319174 | 390243 |  |
| Italy | 150502 | 137734 | 136203 | 145981 | 152807 | 144851 | 153334 | 145797 | 146131 | 176638 |  |
| Spain | 98752 | 95448 | 94255 | 105641 | 108255 | 101483 | 106610 | 102642 | 106000 | 132845 |  |
| UK | 153350 | 143868 | 139339 | 153776 | 162546 | 151529 | 163615 | 189863 | 193976 | 240098 |  |
| **Total drug expenditure (million Euros)** | | | | | | | | | | |  |
| **Cancer** | **2006** | **2007** | **2008** | **2009** | **2010** | **2011** | **2012** | **2013** | **2014** | **2015** | **Source** |
| France | 2589 | 3116 | 3599 | 3385 | 3224 | 2971 | 2987 | 3160 | 3379 | 3833 | IQVIA MIDAS, inflation-adjusted |
| Germany | 1833 | 2156 | 2471 | 2645 | 3941 | 3796 | 3959 | 4595 | 4922 | 5342 |  |
| Italy | 1378 | 1662 | 1850 | 1986 | 2118 | 2167 | 2132 | 2336 | 2532 | 2823 |  |
| Spain | 1188 | 1329 | 1500 | 1698 | 1785 | 1696 | 1593 | 1608 | 1692 | 1918 |  |
| UK | 1158 | 1346 | 1305 | 1260 | 1415 | 1395 | 1562 | 1701 | 2136 | 2752 |  |
| **CVD** |  |  |  |  |  |  |  |  |  |  |  |
| France | 4987 | 5003 | 4771 | 4693 | 4304 | 3993 | 3600 | 3395 | 3227 | 3053 | IQVIA MIDAS, inflation-adjusted |
| Germany | 4528 | 4206 | 4072 | 4115 | 4118 | 3972 | 3775 | 3859 | 3941 | 3970 |  |
| Italy | 3558 | 3575 | 3405 | 3391 | 3276 | 3125 | 2832 | 2774 | 2756 | 2852 |  |
| Spain | 2213 | 2275 | 2313 | 2400 | 2335 | 2063 | 1814 | 1733 | 1686 | 1705 |  |
| UK | 2926 | 2816 | 2097 | 1794 | 1671 | 1417 | 1390 | 1281 | 1418 | 1734 |  |
| **Diabetes** |  |  |  |  |  |  |  |  |  |  |  |
| France | 729 | 774 | 800 | 836 | 931 | 973 | 1017 | 1062 | 1082 | 1089 | IQVIA MIDAS, inflation-adjusted |
| Germany | 1953 | 2023 | 2101 | 2178 | 2264 | 2281 | 2347 | 2418 | 2442 | 2509 |  |
| Italy | 654 | 745 | 830 | 917 | 983 | 1022 | 1060 | 1149 | 1183 | 1258 |  |
| Spain | 406 | 436 | 463 | 541 | 613 | 652 | 676 | 722 | 788 | 867 |  |
| UK | 1128 | 1171 | 973 | 906 | 1009 | 994 | 1050 | 1023 | 1151 | 1411 |  |
| **Neurological disorders** | | | | | | | | | | |  |
| France | 1022 | 1097 | 1135 | 1200 | 1261 | 1257 | 1185 | 1178 | 1174 | 1191 | IQVIA MIDAS, inflation-adjusted |
| Germany | 1716 | 1877 | 2162 | 2460 | 2644 | 2672 | 2689 | 2784 | 2960 | 2761 |  |
| Italy | 623 | 664 | 718 | 795 | 872 | 889 | 896 | 917 | 952 | 1015 |  |
| Spain | 742 | 822 | 883 | 982 | 1046 | 1080 | 1043 | 1040 | 1082 | 1075 |  |
| UK | 894 | 1002 | 902 | 846 | 917 | 972 | 1075 | 1102 | 1295 | 1696 |  |
